# DDT and Breast Cancer Revisited: New Findings in an Old Debate

**DOI:** 10.1289/ehp.115-a505b

**Published:** 2007-10

**Authors:** John Manuel

Author Rachel Carson made the initial claim in her 1962 best-seller *Silent Spring*. Subsequent studies, including a meta-analysis published in *EHP* [López-Cervantes et al. 112:207–214], downplayed the connection. Now researchers have found new evidence potentially linking DDT with breast cancer in women **[*EHP* 115:1406–1414; Cohn et al]**. The findings come at a time when DDT is once again being promoted as a tool to combat malaria.

DDT was widely used as a pesticide in the United States and elsewhere beginning in the mid-1940s. Although DDT was highly effective in reducing the incidence of malaria, scientists began to suspect it was damaging to the environment, specifically to birds of prey in which the DDT metabolite DDE caused thinning of eggshells. Reacting to a growing fear of pesticides, some suspected a link to human cancer. In the early 1970s, DDT was banned for virtually all uses in the United States and in many other countries around the world.

Numerous chemical substitutes for DDT have been developed over the years, but few are as cheap or effective at controlling malaria as DDT. At the same time, most studies conducted since the ban have typically failed to establish a link between exposure to DDT and human cancer. Weighing the perceived health risks against the possible benefits, the WHO and other agencies have recently endorsed the indoor spraying of DDT in areas with high rates of malaria.

But the authors of the current study believe it is premature to suggest there is no link between DDT and human illness, specifically breast cancer in women. They observed that earlier studies were limited by their inability to consider subjects’ age at the time of exposure or measure exposure at a young age during the time DDT was in widespread use. Based on animal studies showing that early exposure to toxicants is often most strongly associated with illness, the authors hypothesized that women who were exposed to DDT in childhood or adolescence might show a higher evidence of breast cancer than the general exposed population.

The authors analyzed the serum of women who sought obstetric care between 1959 and 1967. These women had donated blood as part of the Child Health and Development Studies. The mean age of the women was 26 years. Most would have been 4–12 years old during the period 1945–1959, when DDT was in widest use in the United States.

Researchers analyzed 129 case–control pairs, cases being defined as women subsequently diagnosed with breast cancer before age 50. The study found that high serum concentrations of *p,p*′-DDT, the primary component of DDT, predicted a fivefold increased risk of breast cancer among women who were born after 1931. Women who were born in 1931 or earlier showed no increased risk of breast cancer.

Based on these findings, the authors conclude that it is too early to decide that DDT exposure has little public health significance for breast cancer. They state that many women in the United States who were exposed to DDT in their youth have not yet reached age 50, the age above which women have the greatest risk of evidencing breast cancer.

## Figures and Tables

**Figure:**
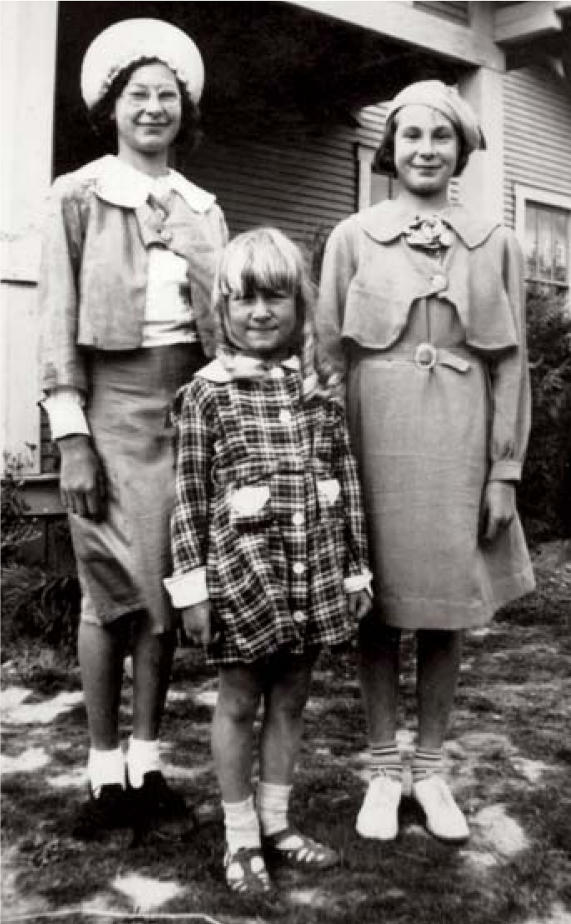
Exposures gone by A new study asks whether women exposed to DDT in childhood, during the height of the chemical’s use in the United States, may now be at greater risk for breast cancer.

